# Homeland, emotions, and identity: Constructing the place attachment of young overseas Chinese relatives in the returned Vietnam-Chinese community

**DOI:** 10.3389/fpsyg.2023.984756

**Published:** 2023-02-02

**Authors:** Zhangwen Shu, Yuan Du, Xuzhou Li

**Affiliations:** ^1^School of Sociology and Anthropology, Xiamen University, Xiamen, China; ^2^School of Education, Chuxiong Normal University, Chuxiong, China; ^3^Faculty of Education, Yunnan Normal University, Kunming, China

**Keywords:** young overseas Chinese relatives, place attachment, qualitative analysis, daily life, cultural system approach, Confucian cultural system

## Abstract

Little attention has been paid to the place attachment and homeland construction for refugees and their descendants in China. This study investigates the process by which the place attachment of Young Overseas Chinese Relatives is shaped in the context of resettlement sites. This qualitative research employed ethnographic fieldwork, and the author collected local literature and materials from February to December 2019 through participatory observation, in-depth interviews, and questionnaires. It is believed that the construction of a new homeland in the community, the emotional experience of the Young in childhood, and the cultural logic of place attachment shape place attachment. The process by which place attachment is shaped is interwoven with homeland construction, which indicates that the living state and mentality of the Young are becoming increasingly stable. The Young developed different mentalities on the basis of traditional Confucian culture in responding to the socio-cultural environments. The resettlement site has become a homeland to which young persons are solidly attached, people give this site meanings and experience certain emotions regarding it, which generates place identity and begins the process of homeland construction.

## Introduction

Home is an exemplary kind of place where people feel a sense of attachment and rootedness. Home, more than anywhere else, is seen as a center of meaning and field of care (Cresswell, 2014). Generally, people are attached to their homelands. When people invest their emotions in their homeland but are forcibly driven out of those lands by external forces, the parties’ resting emotions may be complex. In 1978 the Vietnamese authority deliberately allowed its Chinese citizens to leave the country because of the deteriorating relations between Vietnam and China (Lam, 2000). Many more overseas Chinese fled to China, they were mainly from northern Viet Nam, where they had lived for decades, and they were mostly fishermen, artisans and peasants (Cutts, 2000). Since that time, many refugees from Indochina have flocked to Southeast Asia as well as other countries and regions, thus leading to an international disaster during the twentieth century.

The Chinese Government assisted the refugees upon their arrival; later, they were officially recognized as refugees and resettled to six southern provinces in China (Song, 2014). China subsequently established a project to settle the refugees (Cutts, 2000). It has been more than 40 years since these persons returned to China. Initially, these locations were merely resettlement sites, but they have become a homeland, and resettlement sites have become a vital location for exploring place attachment.

Place attachment is the core concept of place theory; therefore, many researchers believe that place attachment emphasizes the emotional connection between people and places. Humanistic geographers argue that a bond with a meaningful space, or sense of place is a universal affective tie that fulfills fundamental human needs (Scannell and Gifford, 2010). Tuan (1990) uses the term ‘topophilia’ to refer to place attachment, the word “topophilia” is a neologism, useful in that it can be defined broadly to include all of the human being’s affective ties with the material environment. More permanent and less easy to express are feelings that one has toward a place because it is home, the locus of memories, and the means of gaining a livelihood.

For many individuals, childhood place experience plays an essential role in adult identity (Cobb, 1977; Marcus, 1992). Morgan (2010) found that place attachment over the entire human life span found that feelings of connection or belonging to a place increased as people aged and that place attachments formed in childhood were more robust than those formed later in life. Strong bonds to place are only possible when individuals remain in their place of origin for the duration of childhood. This finding reflects a widespread agreement in the literature that the foundations of place attachment are laid down in middle childhood. Robert Hay (1998) found that the development of a rooted sense of place was most influenced by the following factors: length of residence, being born and/or raised in the place, having family and/or close friends living in the place, owning your own home there and being involved in the community. Some studies proposed that the long-term experience of an individual of the physical and social aspects of a place (Ramkissoon et al., 2018; Ramkissoon, 2020), such as biology, environment, psychology, and sociocultural context of the place, develops their place attachment (Shang and Luo, 2021). As introduced above, researchers generally regard emotional factors as an essential connotation of place attachment, and place experience in childhood can affect place identity in adulthood. However, few studies have been able to consult any systematic research concerning the ways in which childhood place experiences shape place attachment.

This study explores the influencing factors and construction process associated with the place attachment of Young Overseas Chinese Relatives in terms of three aspects: childhood local memory, daily life, and emotional experience of young persons. This research can help us understand the lifestyles and psychological states of the Young in such a situation and contributes to the exploration of the cultural adaptation mechanism employed by these individuals. At the practical level, this research is conducive to clarifying the true attitudes and emotions of young persons regarding each resettlement site and subsequently predicting their home-building actions in the resettlement site, which has practical significance for promoting interaction between the resettlement site and local society.

## Research objects and methods

### Selection of research subjects

For our study, we selected Young Overseas Chinese Relatives in Ganba Community, Ganzhuang Sub-district, Yuanjiang, Yunnan Province (formerly Ganba Branch of Ganzhuang Returned Overseas Chinese’ farms). The ancestral home is in Fangchenggang, Guangxi Zhuang autonomous region. In this study, Young Overseas Chinese Relatives (hereinafter, the Young) refer to persons within the scope of children, brothers, sisters, and grandchildren of returned overseas Chinese, who generally live in the resettlement site. During our field investigation, we found that the oldest person born at the resettlement site was 41 years old. Therefore, this study focused on the ages 18–41 years. By the end of 2018, the total number of returned overseas Chinese and their relatives was 2,441. Among them, there are 820 Young Overseas Chinese Relatives, 425 males and 395 females.

From returned overseas Chinese’ perspective, as they were expelled by the Vietnamese authorities, their painstakingly managed homes (Vietnamese residences) were destroyed. After returning to China, they were relocated to various farms reserved for returned overseas Chinese, and they faced the practical problems of rebuilding new homes. When returned overseas Chinese came to the resettlement site, the relationship between the people and the place changed significantly. Ganba was incorporated into the Returned Overseas Chinese farms’ system from the previous people’s commune. The critical impact was that the local spatial pattern, ethnic composition, and livelihoods changed. The reality of the homeland and this series of changes and tribulations have made the resettlement sites an essential research field for investigating the place attachment of returned overseas Chinese and their relatives.

The shaping of place attachment is inseparable from the accumulation of daily life, especially the returning immigrant groups. Only when they use the resettlement site as their new homeland will strong place attachment arise. The construction process of the place attachment of the Young is closely intertwined with the process of the new homeland. By discussing their place attachment, the future development trend of the new homeland can be clarified. The choice of the Young is mainly based on the notice by the Overseas Chinese Affairs Office of the State Council Issuing the Provisions on Defining the Identities of Overseas Chinese, Chinese of Foreign Nationalities, Returned Overseas Chinese, and Relatives of Overseas Chinese [No.5 (2009) of the Overseas Chinese Affairs Office of the State Council].

### Field investigation

Phenomenology, hermeneutics, and critical theory have been employed in place research, but only two are considered in-depth here: psychometrics1 and phenomenology (Patterson and Williams, 2005). Williams and Vaske’s (2003) place attachment scale is one of the first validated scales to systematically identify and measure meanings over a variety of land use settings. It is based on the place attachment constructs of place identity and place dependence (Brown and Raymond, 2007). The strength of place attachment is predicted by certain social and demographic factors, one of which is owning one’s home. People who own their own homes have invested in their local areas, making it likely that they will live there in the long term, which is also a predictor of place attachment and place identity (Anton and Lawrence, 2014).

Although quantitative research can reveal the importance and emotional intensity of different groups of people in relation to “place,” it is difficult to answer the question, “What is the meaning of a place to people.” This qualitative research employed ethnographic fieldwork, it is worth mentioning that, little attention has been paid to the place attachment and homeland construction for refugees and their descendants in China. Despite their important role in building their homeland as well as in community development, their voices are less often heard. The study of place attachment is inseparable from investigating the geographical, cultural, and political environments in which a research object is located. Ethnographic fieldwork, provides profound insights into the role of emotion in social life (Beatty, 2014). The basic idea of ethnographic fieldwork is to give an outline of the social constitution, and disentangle the laws and regularities of cultural phenomena from the irrelevances. One of the key components of the fieldwork is gathering specific data from a variety of facts (Malinowski, 1992). Adheres the holistic view of cultural anthropology, and focuses on the interconnections and interdependence of all aspects of the human experience in all places and times—both biological and cultural, past and present (Haviland et al., 2010).

The authors synthesized three data collection methods and adopted an ethnographic writing style. To analyze the influencing factors and construction process of the place attachment of the Young, the author collected local literature and materials from February to December 2019 through participatory observation, in-depth interviews, and questionnaires:

1. Participant observation is the foundation of cultural anthropology. It produces the kind of experiential knowledge and produces effective, positivistic knowledge, participant observation turns fieldworkers into instruments of data collection and data analysis. During the field investigation, we participated in the daily life of young overseas Chinese relatives. First, we made a more detailed description of the life rhythm, habitual behavior, and daily work of the Young. We gained insight into their lives by harvesting mangoes with them, eating together, and walking together, all of which are described in our interview materials. For example, we got these memories and emotional experiences of young persons’ childhood through face-to-face conversations and observation. On the other hand, we participated in festivals and major ceremonies during the year, and conducted relevant interviews. Where it was necessary to record the ceremonial process, video recordings were made after obtaining the consent of the parties, so as to collect qualitative data. This method made it possible to track the emergence of these attitudes and behaviors and to tie them to likely causes.
2. In-depth interviews provided valid data on the emotional experience of the Young and the cultural logic of the place attachment. In particular, the emotional experience of fear and attachment to the resettlement site in their childhood. Some findings reported in this essay are based on 30 in-depth interviews with youth. Respondents were selected based on prior knowledge and snowball sampling techniques to ensure that these interviews were representative and comprehensive. Participants were from three villages, all born in Ganba resettlement site. The length of time participants lived at Ganba resettlement site ranged from 18 to 41 years (*M* = 29.5 years), 19 males and 11 females. Data collection ceased after 30 interviews when no new themes were developing from the data, indicating that data saturation had been achieved (Guest et al., 2006). This is an appropriate sample size for thematic analysis which anticipates a minimum of 6–10 participants (Braun and Clarke, 2013).
3. Questionnaires are similar to structured interviews, it is that each respondent has to hear exactly the same question. We use the questionnaire method as a complement to in-depth interviews to help clarify potentially ambiguous items. The findings reported in this essay are based on 30 semi-structured interviews with residents of the two regions. Interviewees were selected on the basis of prior knowledge and through a snowball sampling technique that aimed to ensure diverse representation. We refer to Boğaç’s questionnaire, the questionnaire consists of four open-ended questions designed to probe the general characteristics of Young Overseas Chinese’ attachment to their homeland. The questions included:

“Are you happy with your house? (Why).”

“Can you call this place ‘homeland’?”

“Does the resettlement site meet your needs?”

“Where would you like to live in the future? (Why?)”

## Constructing place: The spatial basis for the place attachment of the young

The process by which place attachment is constructed is closely related to the notion of “placemaking.” Especially with respect to the immigrant group of returned overseas Chinese individuals from Vietnam, the Ganba resettlement site was initially an unfamiliar space. These individuals did not establish any social relations or emotional connections in this location. However, after the returned overseas Chinese individuals had lived in the resettlement site for more than 40 years, the most significant factor in the relationship between the people and the place is that the Ganba resettlement site has changed from an unfamiliar space to a new homeland for these returned overseas Chinese individuals. The Young were born and raised at the resettlement site, and they thus experienced the process by which the resettlement site was developed into a new homeland.

From the perspective of place theory, the process of new homeland construction belongs to place construction, just as Lefebvre (1991) said: it is the process of the daily life of new production relations and social relations, the most fundamental of which is around livelihoods. A series of production and consumption activities are carried out, which guarantees the local material basis and constitutes the basis of social relations. This means that the construction of material space is significant in redefining and dividing space in the global political economy, which involves the impact of social, political, and economic factors on people’s usual geographical environment.

The construction of the new homeland for returned overseas Chinese is directly related to the government’s resettlement policy and administrative management. China’s policy to Indochinese refugees is the result of Vietnam’s anti-China and Chinese exclusion action and China’s accommodation of the refugees (Zheng, 2015). The government’s basic guidelines were: to settle in rural areas instead of cities; to allow large clusters; and to assign employment in accordance with skill and experience (Lam, 2000). The farm is a place that is constantly constructed under the state’s leadership through the relationship between the people and the place generated by resettlement, production policies, farm administration, and spatial planning. Especially, the policy of “centralized resettlement is the mainstay, and decentralized resettlement is supplemented” makes returned overseas Chinese generally resettled in various returned overseas Chinese farms.

In 1978, the Ganba resettlement site was first established as the Ganba branch of the Ganzhuang farm for returned overseas Chinese individuals. At that time, there were two sources of resettlement funding: the Chinese government and relevant international organizations. The Chinese government has since allocated more than 100 million yuan to the reception and resettlement of such individuals in Yunnan Province. The government constructed houses, reclaimed land, purchased cattle, horses, and other large livestock, provided tools for production and daily life to returned overseas Chinese, and solved the emerging cultural, educational, medical, and health problems.

In the early days, when returned overseas Chinese were resettled on their farms, the farm was responsible for production and construction. In Returned Overseas Chinese farms, production and living are relatively concentrated. The farm allocates land and other production materials in a uniform manner. After adapting to the local natural conditions, the farm has formulated a business policy of “food self-sufficiency and the vigorous development of sugar cane.” Anyone who can work can become a farmer. Initially, the land cultivated by each employee was allocated by the production team to which they belonged. Employees were not allowed to decide freely which crops to plant on cultivated land. They were required to grow sugar cane and supply their crops to the sugar factory associated with the farm.

Returned overseas Chinese choose to live on these farms, which means that their livelihoods are directly intervened by the farms. Their economic income mainly comes from sugarcane cultivation. The stability and convergence of their livelihoods have laid a material foundation for the farm to become a new homeland. Since then, the farm has gradually developed into a community that can meet the production and living needs of returned overseas Chinese, and their lives are guaranteed. In 1985, the contract responsibility system was implemented on the farm, and the farm set the standard of “220 days for men and 200 days for women,” and if the workers failed to meet the required working hours, they were treated as temporary workers. By 2000, the Ganzhuang Returned Overseas Chinese farm began to change its original plan and control of the state-owned agricultural and forestry farm and established a management system suitable for the market economy. After comprehensive market conditions and actual local conditions, they chose to replace sugarcane planting with mango planting.

For more than 40 years, returned overseas Chinese and their relatives who took root on the resettlement site gradually developed commonality in terms of economic income and psychological quality, and the site became their new homeland, which created place attachment for the Young, making development possible. As witnesses to the construction of the homeland of the returnees, some Yi people state this:

I think in the past, the returned overseas Chinese were not as industrious as Yi people, they used to take a teapot and drink tea when they worked, as if they were going to play in the field, and they went home early to rest after working for two or three hours. But in the past few years, they have started to work hard, and they have invested much money in mango planting. In the past, the returned overseas Chinese did not decorate their resettlement houses much, much less intend to build new houses, and probably live here temporarily. In the past few years, new houses have been built, and they have opened a lot of restaurants and stores, which means they are going to settle down here.

## The shaping of the place attachment of the young in the daily life of childhood

People’s perception of place is inseparable from “lifeworld,” Seamon (2000) believes that the lifeworld refers to the tacit context, tenor, and pace of daily life to which normally people give no reflective attention. One significant dimension of the lifeworld is the human experience of place, which continues to be a major focus of phenomenological work in environment-behavior research. The phenomenologists call for a return to the everyday lifeworld of lived experience (Dovey, 1985) and a move away from the objectification of place and its meaning (Million, 1992).

Complex and diverse emotional relationships emerge between people and places in daily life. Faced with various places, people tend to exhibit different emotional experiences. In particular, there are two kinds of emotional experiences that primarily occur in the interaction between young persons and resettlement site during childhood: the emotional experience of fear and the emotional experience of place attachment.

### Emotional experience: The transition from fear to attachment

Initially, when the returned overseas Chinese individuals began to live at the resettlement site, they faced an unfamiliar geographical environment and thus had an emotional experience of fear. This emotional experience is closely related to the rumors encountered by the Young as well as to their cultural logic. Belief in the existence of ghosts led to a sense of insecurity, and the returned overseas Chinese individuals passed this information to the children, which in turn caused the children to have frightening emotional experiences.

When the returned overseas Chinese individuals were initially resettled in this location, there was a clinic in the area. These individuals believed that the clinic was a place where people could be treated and saved. It was inevitable that some people would bleed, suffer and die during the process of treatment. One young man recalled this situation as follows: “When I was a child, our parents often told us not to go out to play after dark. According to my father, a long time ago, there was a clinic here, and those who fought, the injured people were treated in the clinic. If they died accidentally, they would be buried nearby. Parents were worried that our children would see unclean things, so they did not let us play around.” Therefore, young persons felt that the area where the clinic was located was unclean and might be inhabited by ghosts.

In addition, these individuals also reported fearful emotional experiences pertaining to ponds and reservoirs, which they believed might contain water-related ghosts waiting for people to drown. Their parents rarely let children play alone by the pond to prevent drowning. Such information is widely disseminated in the resettlement site. One of the results is that there is fear in the childhood spatial memories of the Young. A 38-year man recalled:

When we were young, our parents didn’t let us go too far alone. We usually only played near home, and we were afraid when we went far. We only dared to go to the ditch not far from home to play because we were familiar with it, but when we went to the ditch to play, we had to bring a group of children to feel safe.

Returned overseas Chinese and their relatives overcome the fear of living on the resettlement site and gain a sense of security through the use of cultural practices such as folk beliefs and ancestor worship. During the field investigation, we discovered the following cultural practices:

First, the worship of Huagong and Huamu is a typical cultural practice. To returned overseas Chinese, Huagong and Huamu are gods who protect the healthy growth of children. Generally, children do not need to enshrine Huagong and Huamu if they are healthy and safe. Only when a child is often sick or crying at night do parents ask the local master to view the Bazi (Eight Characters of Birth) and give the child a nickname. Generally, a shrine is placed in the room where the child sleeps. If the child cries in the middle of the night in the future, the parents will come to the shrine to burn incense, praying to Huagong and Huamu to take care of the child. It is worth mentioning that the cultural connotations and ritual behaviors of the folk beliefs of the Huagong and Huapo are closely related to the ancestral home (Guangxi Zhuang autonomous region) of returned overseas Chinese (especially in the Fangchenggang city), which means that they overcome fears by actively using the cultural traditions from the ancestral home of Guangxi.

Second, soul-calling is another cultural practice that aims to cause children who are frightened and who have lost their souls to return to normalcy. If a child turns pale and becomes fidgety after having been frightened, parents may attribute these phenomena to the child’s loss of soul. When calling the soul, the ceremony host carries the child’s clothes in his left hand alongside a bowl of rice and an incense stick in his right hand and then travels to the place where the child’s soul was lost. Subsequently, he throws the rice around and shouts “our child, come back.” The child, who is present, says “back in the body, back in the body.” The child is then asked to put the clothes on their bodies. The next day, the child returns to normal.

Third, seeking the blessing of ancestors. Returned overseas Chinese have a strong sense of ancestor worship. When they encounter disasters, they seek the blessing of their ancestors. A 19-year-old man said:

When I was in middle school, I had a good friend. One day he drowned while swimming in a pool. One night after his death, I saw him in a dream. I was invited to go swimming, but the horror was that I saw a female ghost floating around him. I felt my soul was gradually separated from my body. At this point, an older man with white light appeared beside me. The older man slowly pushed my soul back into my body; then, I woke up with a start. The next day, I told my grandfather about this dream. My grandfather said that it might be my great-grandfather who came to protect me; so, my grandfather taught me to burn incense in front of the ancestral tablet and pray to my ancestors. Blessed, I haven’t had any similar dreams since then.

Culture-inclusive theories for Confucian morphostasis can be effectively used to explain the behavior of returned overseas Chinese. Hwang (2015b) constructed a series of culture-inclusive theories to integrate findings of previous empirical researches, and conflict resolution in Confucian society. Hwang (2012) indicated clearly that ancestor worship ceremonies in folk society also display the earnest Chinese desire for balance with the supernatural. Under the influence of traditional Confucian culture, returned overseas Chinese and their relatives have developed the Confucian mentality. They often utilize means from traditional Confucian culture to deal with problems in their daily lives, and overcome the fear of living on the resettlement site and gain a sense of security through the use of cultural practices such as ancestor worship and folk beliefs.

It should be mentioned that the emotional experience of fear is not the most common experience of the Young in their childhood. Still, these experiences illustrate people gradually adapting to the resettlement site. The emotional experience of fear gradually weakens as the overseas Chinese adapt to the resettlement site’s environment and cultural practice and finally only stays in people’s collective memory. With the accumulation of time living at the resettlement site, the Young develop new emotional experiences. These experiences are closely related to place attachment and are accumulated and shaped over time.

### Happiness and attachment: Daily life of the young in childhood

The daily life experiences of the Young influence the construction of place attachment, and it is gradually constructed in daily life. Place attachment is also shaped during childhood. The process of the Young constructing place attachment has its characteristics, this study sought to understand the emotional attachment of the Young to a place in their daily life. According to the field investigation, place attachment gradually forms in daily life during childhood and the constant interaction with the resettlement site’s natural and cultural environments. This means that people’s place attachment is established in childhood, and strong place attachment is likely to develop when a person lives in one place for an extended period in childhood.

Happiness is a positive experience that young persons encounter during childhood. They become immersed in their interaction with the resettlement site by playing games in this location. One 38-year-old woman recalled this experience as follows: “When I was a child, I liked to go to the mountains to catch birds and fish in the pond. In spring, I went to the fields to pick wildflowers, and in autumn, I went to the mountains to pick wild fruits. When the sugar cane was ripe, I would ask my friends to play in the field.” It can be seen from this interview that the places in which these individuals played during their childhood are closely related to nature and that they feel a unique sense of place in such an environment. Chawla (1992) believes that children are attached to a place when they show happiness at being in it and regret or distress at leaving it, and when they value it not only for the satisfaction of physical needs but for its intrinsic qualities. In this study, the Young obtained survival skills and a sense of security during their childhood, and they experienced positive emotions such as happiness at the resettlement site.

Generally, place attachment involves people’s expectations and satisfaction with place stability, producing rich local knowledge of the place and behaviors that help maintain or strengthen attachment, thereby enhancing their sense of place—a positive emotional experience. Year-round, these individuals receive gifts from the resettlement site: in spring, when the kapok trees are in full bloom, they follow their parents to pick up the kapok scattered on the ground. In their early years, when they migrated from Vietnam to the resettlement site, they were unfamiliar with the use of kapok and regarded it as worthless. After time passed, they mastered the art of eating kapok, and kapok became a delicacy. On the eve of the Spring Festival, they traveled to the mountains to pick wild phrynium capitatum and engaged in Zongzi worship of their ancestors; in late autumn, when olives ripened, they visited the hills to pick wild olives with their parents and ate olives with chili peppers. The evidence presented thus far supports the idea that these gifts mainly come from the natural environment of the resettlement site, indicating that they have produced a series of local knowledge, which in turn strengthens their emotional attachment.

### Bao Yidi: The cultural logic of the place attachment of the young

The place attachment and identity of young persons are positively related to their understanding of the resettlement site as their homeland. Since they were born and raised at the resettlement site, this place naturally became their homeland. This understanding comes from the cultural logic of Bao Yidi (namely afterbirth). Bao Yidi has a special treatment and cultural connotation for these people. Returned overseas Chinese individuals once had a custom of burying the afterbirth in their houses or in the mountains. Bao Yidi refers to the place where the afterbirth will be buried. This place is where one was born and grew up, and this place has naturally become an emotional homeland. Bao Yidi is an essential criterion for judging whether a place is their homeland and has become a cultural symbol that condenses emotions.

There is the challenge of the discontinuities between pre-and post-migration identities, as well as between the first and second generations. These discontinuities create the experience of watching one’s children grow up with a different sense of place and homeland, and become culturally competent in new environments, even in childhood, often surpassing their parents (Ewing, 2005). The object of place attachment for elders is mainly the place of residence in Vietnam, however, Vietnam is an unfamiliar place for young people. The young follow their elders to visit their relatives in Vietnam, and in the homes of their relatives in Vietnam, they are curious about Vietnam’s local customs. As discussed by this 19-year-old male informant:

The climate in Vietnam is hot and humid, and I was very uncomfortable with it when I first got there. They sit on the floor and eat, and I think they may eat more. They have to shake hands with each other before eating, and I don't know why they do that. The older relatives speak our Hakka Chinese, but young people only speak Vietnamese. When I was chatting with Vietnam's cousin, I needed the help of translation software.

“The resettlement site of Ganba Community is my hometown” is the emotional expression of the Young in reference to Ganba. The opinion of a 20-year-old man is typical:

The household registration is already here, which shows that our roots are already here. I don’t regard Vietnam as my homeland because my roots are here in Ganba Community. According to my grandfather, Vietnam used to be chaotic, so my family moved back from there. Vietnam is a place where I have relatives and a scenic spot, but I have never been to Vietnam. I have only been to Guangxi Zhuang autonomous region. When I go to Guangxi, I feel like going to relatives’ houses because my relatives in Guangxi speak the same vernacular and I feel cordial.

These words show that when the Young define their Bao Yidi, they often compare their ancestral home in Guangxi, their parents’ residence in Vietnam, and the resettlement site of Ganba. They define their homeland based on where they were born and raised and look at their world based on that place. The resettlement site has become the center and foothold of the Young moving in space, even if they leave the resettlement site for work, study, or other reasons, they generally want to return to the resettlement site. A 30-year-old man described the work plan in the Guangdong province thus:

By the fruit harvest season this year, I will sell the mangoes and then graft new mango varieties, and I will work in the Guangdong province. I hope that after three years, I can make some money and return to Ganba Community. The previously grafted mango varieties should have already set fruit, and I can concentrate on managing mangoes at home, and I don’t plan to go out.

A 20-year-old male described his planning in this way:

Ganba resettlement site is my Bao Yidi, and I plan to live here with my family for the rest of my life. There are many jobs I can do here to support my family. In the past few years, I have been engaged in motorcycle maintenance, but this is just a temporary work for me because I have to cultivate the field at least half of the year. I plan to do more temporary work, so that if the mango income decreases in a certain year, then I can still do a part-time job so that the income will be more guaranteed, and we can live better here.

The ultimate goal of the work plan is to make money in other places and then return to the resettlement site so that the family can live a better life. Such plans are precisely the operation of the cultural logic of Bao Yidi.

### “I am from Yuanjiang”: The interactive influence of identity and attachment

According to Proshansky (1978), place identity is a sub-structure of the self-identity of the person consisting of broadly conceived, cognitions about the physical world in which the individual lives. These cognitions relate to the variety and complexity of physical settings that define the day-to-day existence of every human being. At the core of such physical environment-related cognitions is the environmental past of the person; a past consisting of places, spaces and their properties which have served instrumentally in the satisfaction of the person’s biological, psychological, social, and cultural needs. As Wullenkord et al. (2020) put it: Place identity refers more strongly to the cognitive-emotional bond to a place. In this study, the place identity of young persons is closely related to the fact that they regard the resettlement site as an extension of themselves, and “I am from Yuanjiang” has become a way of expressing their place identity.

In contrast, although the elders have lived at the Ganba resettlement site for more than 40 years, they do not identify as natives of Yuanjiang emotionally. One reason is that their Bao Yidi is not a resettlement site; therefore, they call the Yi people and Dai people living around the resettlement site “natives” and call themselves “returned overseas Chinese.” Compared with the elders, during the fieldwork, we observed that Young Overseas Chinese Relatives have developed a unique perception of the resettlement sites, most notably that they believe the resettlement sites have their own “character.” When we conducted fieldwork in the resettlement sites, Young Overseas Chinese Relatives often said to us, “Have you noticed? Each village has its character.” We present a few representative interviews below:

A 20-year woman recalled:

Each village has its character, and our village is not as quiet as other villages, and even a small thing will be talked about for two months. For example, yesterday before I got up, my neighbors were already sitting in front of my house chatting. Afterwards, I learned it was about a young man who bought a car.

A 24-year man recalled:

Very few people in our village drink alcohol, unlike the people in the other two villages, the Young in the other villages often gather together. But the young people in our village are different, we rarely get together.

A 30-year man recalled:

Each village has its character, and our villagers do not work as hard as other villages. Many young people do not go to work and play games at home all day. In recent years, more and more people are doing business, but the elders often reject our young people. In recent years, young people in other villages have started doing business, but our elders often deny our ideas. They want us to continue growing mangoes instead of doing other work.

From the above interviews, it is clear that the young tended to give an impression of the three settlements, but these impressions were not significant differences between the settlements. The main reason for this phenomenon can be attributed to the cultural logic of Bao Yidi. It is also the tendency of the Young to assign a specific “character” to the three settlements and thus to subdivide them. Hwang (2012) believes that in order to view a person as an agent-in-society, the ways in which the individual follows a certain moral order, takes action, or reacts to others’ actions in systems of social relationships should be investigated. Under the influence of the cultural logic of Bao Yidi, the Ganba resettlement site has become the deepest object of place attachment for young persons, which in turn prompts them to develop a place identity to Yuanjiang County.

The concept of mindsponge was first developed by Quan-Hoang Vuong and Nancy K. Napier in early research articles about acculturation and global mindset (Vuong et al., 2022). Coined by Vuong and Napier (2015), the term mindsponge mechanism describes how an individual absorbs and integrates new cultural values into one’s own set of core values. Overall, the mechanism centers on five components: the mindset, comfort zone, multi-filtering system, cultural and ideological setting, and cultural values (Jin and Wang, 2022). The Young acquire the ability to perceive the world from the Confucian cultural tradition and cope with the issue of place identity. This is a personal need to construct a place identity, because when they mention that “the resettlement sites have their character,” they only compare the three settlements, but not the Yi villages adjacent to the settlements. The fact that the Young have established place identity with the settlement site indicates that the Confucian idea of *the Superior Man loves people as people and cares about creatures* (仁民而愛物) plays a role in the process of homeland construction, that is, the Young see the settlement site not only as a geographical location but also as an important ethical object, and the Young have a moral obligation to the settlement site. Ultimately, the Young develop a strong emotional bond with the resettlement site.

This endorsement of the exclamation “I am from Yuanjiang” manifests in a variety of ways, such as through identification with the local language and diet. In terms of language use, the young have mastered the Yuanjiang dialect (Southwest Mandarin), and they frequently use the Yuanjiang dialect in daily life. Even individuals who do not speak the Yuanjiang dialect very often after leaving, in situations such as when they visit Yuanjiang County, tend to take the initiative to use the Yuanjiang dialect and identity themselves as Yuanjiang people. In terms of diet, they appreciate the local food and cultural symbols, with rice-flour noodles and peppers as the best representatives of this phenomenon. Their elders seldom eat rice noodles and peppers, while the Young regard these delicacies as essential foods.

These individuals did not establish any social relations or emotional connections in this location. However, after the returned overseas Chinese individuals had lived in the resettlement site for more than 40 years, the most significant factor in the relationship between the people and the place is that the Ganba resettlement site has changed from an unfamiliar space to a new homeland for these returned overseas Chinese individuals. The Young were born and raised at the resettlement site, and they thus experienced the process by which the resettlement site was developed into a new homeland.

The construction of a new homeland accompanies the place attachment construction. They put energy and finances into constructing resettlement site, and especially in the last decade, the investment has increased significantly. For example, in 2014, parts of the wasteland of the Young were included in the comprehensive development project of low hills and gentle slopes. During the land acquisition process, parts of the wasteland were requisitioned. After receiving compensation, they chose to build new houses not far from the resettlement site. In addition, they have invested large sums of money in planting crops. Furthermore, they have partnered to install ground drip irrigation and used excavators to convert steep wasteland into terraces. This series of actions shows that place attachment plays a driving role in constructing places.

## Discussion

With the resettlement of returned overseas Chinese to farms in 1978, all the resettlement sites, including the Ganba resettlement site, have built new homelands. This study claims that the construction of a new homeland, the emotional experiences encountered during childhood, daily life, and the cultural logic of place attachment all shape the place attachment of the Young. In the early days of resettlement, the Ganba resettlement site was a relatively concentrated area focused on living and production. The livelihood of individuals living in this location was mainly crop cultivation, and returned overseas Chinese individuals lived a similar life. This experience with the resettlement site and the resulting social status establish a new homeland for returned overseas Chinese and their relatives.

Psychologist Hwang (2005) indicated that indigenous compatibility can be regarded as a guiding principle for the indigenization movement of psychology. Hwang (2015a) keenly indicated that the construction for culture-inclusive theories has to follow a basic principle of cultural psychology: “one mind, many mentalities; universalism without uniformity” (Shweder et al., 1998). This study found that the living state and mentality of the Young are becoming increasingly stable. The resettlement site has become a homeland to which young persons are solidly attached. The Young have progressed from experiencing a feeling of fear to the attainment of safety and happiness. This change has laid a certain psychological foundation for the generation of place attachment and formed a framework that can help maintain and strengthen place attachment. Returned overseas Chinese and their relatives identify the place in which they live with the cultural logic of Bao Yidi, and the resettlement site provides a guarantee of life for them and thus produces a place identity and puts this identity into practice with respect to the construction of a new homeland.

The information absorption process is contingent on the mindset (Nguyen et al., 2022). In the process of constructing place attachment, the Young developed different mentalities on the basis of traditional Confucian culture in responding to the sociocultural environments. Hwang (2012) believed that people living in different societies may develop different mentalities on the basis of this deep structure in responding to their sociocultural environments. For example, the ethics standard of Rén (仁, benevolence) promoted by Confucianism plays an important role in shaping the place attachments of the Young. This ethics standard sees the world as an organic community, and the natural world is a link in the ethical chain of human beings, so man has a moral obligation to the natural environment. Influenced by traditional Confucian culture, returned overseas Chinese and their relatives are concerned with family maintenance and ethical order, following the principle of patrilineal succession and living according to the patrilocal residence. These principles, which are characteristic of traditional Confucian culture still play a role in homeland construction. For example, in terms of residence distribution, most of the returned overseas Chinese who lived in resettlement site have a relative relationship. As a result, relatively homogeneous communities have emerged in which social networks and interpersonal relationships have developed.

In the mindsponge process how individuals to spot and integrate new values as well as reason the values’ appropriateness and usefulness in comparison to the existing core values are underlying themes (Vuong and Napier, 2015). The formation of place attachment to the resettlement site is not an instantaneous process; rather, according to the mindsponge mechanism, it involves a gradual and incessant information processing that the Young adapt to the geographic and socio-cultural environment of the resettlement site by accepting or rejecting new information and values. Our findings indicated that, after the returned overseas Chinese individuals had lived in the resettlement site for more than 40 years, they gradually established social relations in the resettlement site, especially when they intermarried with the local residents, they are able to integrate into the place and inherit traditional Confucian culture. In this case, the resettlement site becomes the new homeland of the Young, which affects their sense of place. Abundant research results indicate that attachment to numerous places continues to be strong (Riethmuller et al., 2021). Boğaç (2009) suggest that participants’ future expectations shaped their attachment to their new homes, while their degree of attachment to their previous environments also played an important role in the attachment process. This case provides a new direction for thinking about cultural adaptation in immigration research and suggests that we should focus on the emotional issues associated with the relationships between people and places.

Although this study is a subject with social application value, it is significant from the point of view of migration, interculturality, and inclusion policies. As with all research, there are potential study limitations that could impact study results. First, the strength of place attachment and the factors influencing it among Young Overseas Chinese Relatives were not well represented in the qualitative data, and needs to use this epistemological strategy to conduct theoretical or empirical research. Hwang (2015a) indicated clearly that the construction of the *Mandala Model* and *Face and Favor* Model are the first step to attain the epistemological goal of indigenous psychology. These models were then used to develop culture-inclusive theories for Confucian morphostasis (Hwang, 2019). We agree with his theory that indigenous psychologists need a new “model of man” which may make a “true cultural turn” to achieve the aim of integrating culture and psychology. Indigenous psychologists have to construct culture-inclusive theories to reflect not only the deep structure of universal human mind but also the mentalities of people in a particular culture (Hwang, 2018).

Second, because this was a cohort study, the findings are by no means representative of all Young Overseas Chinese Relatives in the various resettlement sites. At best, this study can only shed light on the thoughts and feelings of the Young in Ganba resettlement site. Further research should adopt quantitative research, especially scale measurement and related data analysis, to draw relevant conclusions, rather than the more competitive research results proposed in this study. To achieve the goal of developing global psychology, it would take a large-scale research program to travel across the world to investigate all indigenous psychologies, and take into account the history and even the future of each culture (Hwang, 2005). Following this approach, a series of culture-inclusive theories can be constructed to constitute the scientific microworld of Confucian relationism (Hwang, 2019).

Third, the mindsponge framework has effectively explained many psychological phenomena or cognitive shifting processes. As a conceptual framework for information processing, mindsponge can be applied to many human psychological phenomena, consisting of both individual and collective/social levels (Vuong et al., 2022). In this study, the mindsponge mechanism provides insightful explanations for examining the process of shaping local attachments among the Young, and the mechanism helps to explain well the connections of childhood local memory, daily life, and emotional experience of young persons with place attachment. Future studies examining the psychological processes and formation of place attachment could be done based on Bayesian Mindsponge Framework analytics—a framework for a new perspective of the human mind’s information processing mechanism (Vuong et al., 2022).

## Data availability statement

The original contributions presented in the study are included in the article/supplementary material, further inquiries can be directed to the corresponding author.

## Ethics statement

Ethical review and approval was not required for the study on human participants in accordance with the local legislation and institutional requirements. The patients/participants provided their written informed consent to participate in this study.

## Author contributions

ZS, YD and XL participated in the design of this study, they all formulated the hypotheses and contributing to the discussions. ZS and XL carried out the study and collected important background information. ZS employed ethnographic fieldwork. YD and ZS collected local literature and materials. ZS and YD drafted the manuscript. ZS and XL performed manuscript review. All authors contributed to the article and approved the submitted version.

## Funding

This research was supported by the Fieldwork Funds for graduate students of Xiamen University (no. 2019GF001).

## Conflict of interest

The authors declare that the research was conducted in the absence of any commercial or financial relationships that could be construed as a potential conflict of interest.

## Publisher’s note

All claims expressed in this article are solely those of the authors and do not necessarily represent those of their affiliated organizations, or those of the publisher, the editors and the reviewers. Any product that may be evaluated in this article, or claim that may be made by its manufacturer, is not guaranteed or endorsed by the publisher.
